# The multifaceted roles of ketones in physiology

**DOI:** 10.1113/EP092243

**Published:** 2025-05-11

**Authors:** Seyed Amirhossein Tabatabaei Dakhili, Kunyan Yang, Magnus J. Stenlund, John R. Ussher

**Affiliations:** ^1^ Faculty of Pharmacy and Pharmaceutical Sciences University of Alberta Edmonton Alberta Canada; ^2^ Alberta Diabetes Institute University of Alberta Edmonton Alberta Canada; ^3^ Cardiovascular Research Institute University of Alberta Edmonton Alberta Canada

**Keywords:** heart failure, ketogenesis, ketone oxidation, ketones, type 2 diabetes

## Abstract

The production of ketones, referred to as ketogenesis, plays an essential role in maintaining energy homeostasis during prolonged fasting/starvation, which primarily stems from its ability to serve as a fuel source to support neuronal ATP production, thereby limiting muscle wasting. Over the years, the field has come to appreciate that ketones are much more than just a fuel source supporting neuronal metabolism, as many other oxidative organs, such as the heart and skeletal muscle, are capable of metabolizing ketones. Furthermore, ketones appear to be an important fuel source for exercising muscle. Beyond supporting ATP production, it is also becoming widely recognized that ketones are powerful signalling molecules, as they serve as ligands for G‐protein coupled receptors and can even modify gene expression via regulating DNA post‐translational modifications. As they play a key role in supporting whole‐body physiology, it is not surprising that perturbations in ketone metabolism can contribute to various pathologies, particularly in relation to cardiometabolic diseases. Some of the strongest evidence supporting the aforementioned statement is seen for both heart failure and type 2 diabetes. Accordingly, we will review herein the multifaceted roles of ketones in supporting whole‐body physiology, while interrogating the evidence to suggest whether modifying ketone metabolism may have a therapeutic role in the management of heart failure and type 2 diabetes.

## Abstract (highlights)

- **What is the topic of this review?**
  This review details the complex roles of ketones in contributing to whole‐body physiology, focusing on their role in supporting ATP production and regulating cell signalling.
- **What advances does it highlight?**
  This review highlights the latest evidence demonstrating that ketones are an important fuel source for organs beyond the brain, that ketones can modify cell signalling, gene expression and inflammation via their actions on G‐protein coupled receptors and other mechanisms (e.g., post‐translational modifications), and that modifying ketone metabolism may have utility in the management of heart failure and type 2 diabetes.

## 1 INTRODUCTION

Ketones comprise β‐hydroxybutyrate (βOHB), acetoacetate and acetone, which were first identified in the urine of diabetic coma patients at the end of the 19th century, leading to ketones being originally viewed as wasteful products of incomplete fatty acid oxidation in the liver (Puchalska & Crawford, 2017; Robinson & Williamson, 1980). Despite these initial derogatory views of ketones, seminal studies in the 1960s in starved human participants demonstrated that ketones become the major fuel supporting ATP production in neurons of the brain, illustrating that ketones are essential to normal physiology (Owen et al., 1967). The metabolism of ketones as an alternative fuel source for the brain during times of limited carbohydrate availability (e.g., prolonged fasting) is now regarded as foundational to human physiology, given that neurons lack the enzymatic machinery required to oxidize fatty acids. Thus, the field's appreciation and knowledge of ketones as an oxidative fuel source to support whole‐body metabolism has greatly advanced since these original discoveries.

Intriguingly, research in the 21st century has also demonstrated that ketones are not just an alternative fuel source during limited carbohydrate availability, but that ketones also have widespread actions in the regulation of cellular signalling (Puchalska & Crawford, 2017). Indeed, ketones may serve as ligands for G‐protein coupled receptors (GPCRs) or even regulate gene transcription. Furthermore, it is becoming increasingly recognized that perturbations in ketone metabolism arise during the progression of cardiometabolic disease (i.e., type 2 diabetes (T2D) and heart failure) and may directly contribute to disease pathology (Lopaschuk & Dyck, 2023; Soni et al., 2024). As such, strategies to correct malfunctioning ketone metabolism are being explored to prevent and/or reverse the pathophysiology of T2D and/or heart failure.

In this review, we describe the multifaceted roles of ketones in whole‐body physiology. This includes their key role in supporting energy metabolism during periods of starvation, the adaptations in ketone metabolism that take place in response to meal ingestion, and their role in supporting increases in energy demand during exercise. Lastly, we also interrogate whether targeting ketone metabolism has therapeutic utility in managing T2D and heart failure (Table 1).

**TABLE 1:** Summary of actions of manipulating ketone levels in animal and human studies.

Model	Intervention	Metabolic Impact	Effects on glucose homeostasis	Effects on cardiac function	Reference
Canines (heart failure [pacing‐induced])	βOHB infusion	Increased circulating ketones	Not determined	Improved cardiac output and LVEF	(Horton et al., 2019)
Humans (heart failure)	Oral (R)‐1,3‐butanediol	Increased circulating ketones	Improved glycaemia	Improved LVEF and stroke volume	(Guldbrandsen et al., 2025)
Humans (heart failure)	βOHB infusion	Increased circulating ketones	Improved glycaemia	Improved LVEF and stroke volume	(Solis‐Herrera et al., 2025)
Humans (heart failure)	D‐βOHB‐L‐1,3‐butanediol	Increased circulating ketones	Not determined	Improved cardiac output, reduced pulmonary pressure	(Gopalasingam et al., 2024)
Humans (obese)	(*R*)‐3‐hydroxybutyl (*R*)‐3‐hydroxybutyrate	Increased circulating ketones	Improved glycaemia	Not determined	(Walsh et al., 2021)
Humans (T2D)	(R)‐3‐hydroxybutyl (R)‐3‐hydroxybutyrate	Increased circulating ketones	Improved glycaemia	Not determined	(Soto‐Mota et al., 2021)​
Mice (heart failure [TAC])	SCOT KO (cardiac‐specific)	Decreased ketone oxidation	Not determined	Worsened LVEF	(Schugar et al., 2014)
Mice (heart failure [TAC])	BDH1 KO (cardiac‐specific)	Decreased ketone oxidation	Not determined	Worsened LVEF	(Horton et al., 2019)
Mice (heart failure [TAC])	BDH1 overexpression (cardiac‐specific)	Increased ketone oxidation	Not determined	Improved LVEF, reduced cardiac hypertrophy	(Uchihashi et al., 2017)
Mice (heart failure [TAC])	SCOT KO (skeletal muscle‐specific)	Decreased ketone oxidation	No change	Improved LVEF, reduced cardiac hypertrophy	(Byrne et al., 2020)
Mice (obese)	SCOT KO (skeletal muscle‐specific)	Decreased ketone oxidation	Improved glycaemia	Not determined	(Al Batran et al., 2020)
Mice (obese)	Pimozide (SCOT inhibitor)	Decreased ketone oxidation	Improved glycaemia	Not determined	(Tabata et al., 2023a)
Mice (T2D)	Pimozide (SCOT inhibitor)	Decreased ketone oxidation	Improved glycaemia	Improved diastolic function, reduced cardiac hypertrophy	(Greenwell et al., 2024)
Mice (T2D [*db*/*db* mice])	D‐β‐Hydroxybutyrate‐(R)‐1,3 Butanediol	Increased circulating ketones	Improved glycaemia	Not determined	(Thai et al., 2021)
Rats (Exercise)	Endurance training	Increased ketone oxidation enzyme expression	Not determined	Not determined	(Winder et al., 1974, 1975)
Rats (heart failure [left anterior descending coronary artery ligation])	Hexanoyl‐hexyl‐3‐hydroxybutyrate	Increased circulating ketones	Not determined	Improved LVEF, reduced cardiac hypertrophy	(Yurista et al., 2021)

## 2 KETONE METABOLISM DURING FASTING/STARVATION

Owing to the inability of neurons to store sufficient amounts of glycogen, the selective permeability of the blood–brain barrier, and the absence of enzymes required for fatty acid β‐oxidation, ATP production in the brain is primarily dependent on the oxidation of exogenous glucose to meet the brain's energy demands (Duran et al., 2019). However, the brain has mechanisms in place to shift its metabolic preferences when glucose becomes scarce. During prolonged fasting, as demonstrated by the pioneering work of Owen et al. (1967), the brain begins utilizing other fuel sources when glucose availability is severely reduced. When obese individuals underwent prolonged fasting for approximately 40 days, it was observed that cerebral concentrations of ketones were significantly elevated. It was further observed that under extreme fasting, the brain shifts its preferences from glucose to ketones (βOHB and acetoacetate) as its primary fuel source, accounting for ∼70% of its energy production while reducing its need for glucose by 30%. This strategic shift minimizes protein breakdown, particularly in skeletal muscle, allowing the brain to generate ATP from ketones in order to preserve its function while maintaining skeletal muscle mass (Sherwin et al., 1975).

Several peripheral tissues exhibit a comparable metabolic switch, distinguished by the notable aspect that these tissues do not solely depend on glucose. During fasting, there is a notable transition from glucose to lipid oxidation, particularly in skeletal muscle and the myocardium (Soeters et al., 2012). This is characterized by an increase in ketones and free fatty acids, which are a direct consequence of the reduction in circulating insulin levels. During fasting/starvation, muscle fibres demonstrate increased expression of enzymes involved in the uptake and metabolism of ketones (Petersen & Shulman, 2018), thus facilitating their use as a substrate to support ATP production and preserving muscle mass as formerly stated.

**FIGURE 1:**
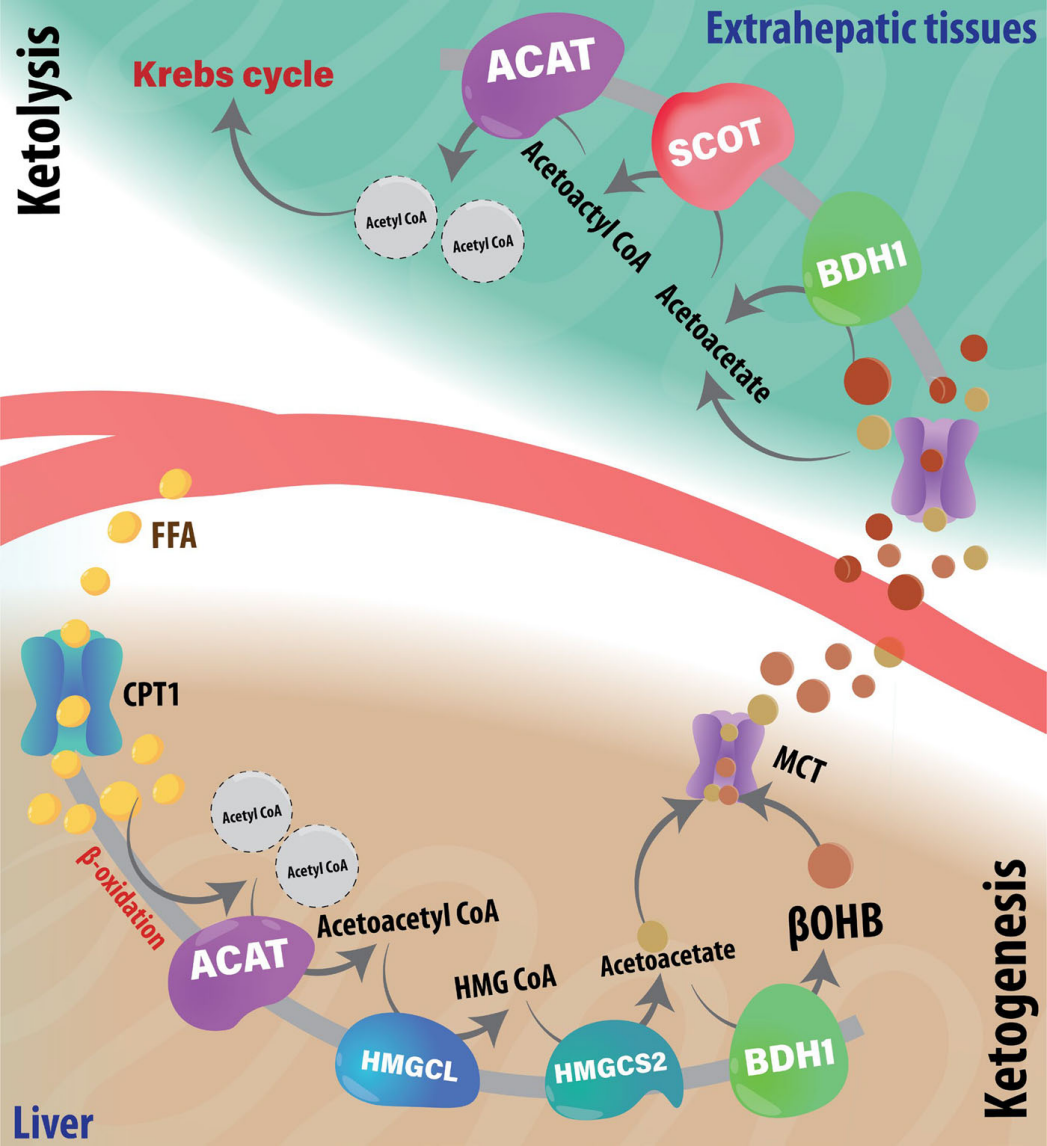
General overview of ketone metabolism. During fasting/starvation/exercise, hepatic fatty acid β‐oxidation generates acetyl‐CoA for ketogenesis. Ketones (acetoacetate, βOHB) are transported to extrahepatic tissues (e.g., brain, muscle, heart), where they are converted to acetyl‐CoA for the Krebs cycle and subsequent ATP synthesis. ACAT, acetoacetyl‐CoA transferase; BDH1, β‐hydroxybutyrate dehydrogenase 1; βOHB, β‐hydroxybutyrate; FFA, free fatty acids; HMG‐CoA, hydroxymethylglutaryl‐CoA; HMGCL, hydroxymethylglutaryl‐CoA lyase; HMGCS2, hydroxymethylglutaryl‐CoA synthase; SCOT, succinyl‐CoA:3‐ketoacid‐CoA transferase.

The stimulation of ketone production in the liver during fasting/starvation, referred to as hepatic ketogenesis, is driven by the mobilization of triacylglycerol stores (lipolysis) in adipose tissue, resulting from decreases and increases in circulating insulin and glucagon levels, respectively. Increased glucagon action activates hepatic peroxisome proliferator activated receptor‐α, which increases expression of the mitochondrial β‐oxidation enzymatic machinery, allowing the liver to increase fatty acid oxidation‐derived acetyl‐CoA (Longuet et al., 2008). While increased acetyl‐CoA production from fatty acid β‐oxidation often shuttles to the Krebs cycle to generate reducing equivalents for the electron transport chain and subsequent oxidative phosphorylation (Puchalska & Crawford, 2017), oxaloacetate levels cannot keep pace, and the excess acetyl‐CoA is diverted to ketogenesis (Figure 1). The diminished supply of carbohydrates during fasting/starvation contributes to the latter, decreasing overall acetyl‐CoA levels due to the reduction in glycolytically derived pyruvate available to enter the mitochondria for decarboxylation to acetyl‐CoA (Masoro & Felts, 1958). Instead, acetoacetyl‐CoA thiolase (ACAT) catalyses the condensation of two acetyl‐CoA molecules (primarily derived from fatty acid β‐oxidation) to form acetoacetyl‐CoA, following which, in the presence of another acetyl‐CoA, 3‐hydroxymethylglutaryl‐CoA (HMG‐CoA) synthase converts acetoacetyl‐CoA to HMG‐CoA. HMG‐CoA lyase subsequently catalyses the conversion of HMG‐CoA into acetoacetate by HMG‐CoA, which can spontaneously decarboxylate to acetone, but is primarily reduced by βOHB dehydrogenase (BDH1) to βOHB, the most abundant ketone in the circulation (Puchalska & Crawford, 2017).

Circulating ketone levels will often range anywhere from 100 to 250 µM and are under circadian regulation. However, in pathophysiological environments where hepatic ketogenesis is left unchecked (e.g., type 1 diabetes), ketoacidosis can arise and is often defined as circulating ketones being greater than 7 mM, though levels can reach greater than 25 mM (Puchalska & Crawford, 2017; Robinson & Williamson, 1980). It is not only the circulating ketone levels that need to be considered, but also the drop in blood pH (∼7.4 in healthy humans) that indicates ketoacidosis. Despite studies demonstrating that circulating ketone levels can reach levels approaching ∼7 mM in animals and humans either fed ketogenic diets or provided ketone ester supplements, this is often referred to as a ‘nutritional ketosis’ and not a true ‘ketoacidosis’ due to blood pH being maintained.

## 3 KETONE METABOLISM IN RESPONSE TO NUTRIENT INGESTION

The consumption of nutrients during food intake triggers major shifts in ketone metabolism, via actions impacting pancreatic hormones and catecholamines, as well as molecular changes at an organ level. Elevations in circulating glucose levels following ingestion of a high carbohydrate containing meal promote islet β‐cell insulin secretion, which stimulates glucose utilization in peripheral tissues (Petersen & Shulman, 2018). Furthermore, insulin is a potent suppressor of adipose tissue lipolysis through its actions to inhibit hormone sensitive lipase, which lowers circulating fatty acid levels and subsequent delivery to the liver, thereby decreasing ketogenesis. Interestingly, studies have suggested that in the presence of a high carbohydrate containing meal, circulating ketones can remain elevated when medium‐chain triacylglycerols are consumed (Vandenberghe et al., 2020). Reasons for this likely reflect that medium‐chain fatty acids are not regulated by malonyl‐CoA and the carnitine palmitoyltransferase‐1 axis (Jong‐Yeon et al., 2002; Sidossis et al., 1996), which would bypass insulin's actions to activate acetyl‐CoA carboxylase and malonyl‐CoA formation, thereby restricting long‐chain fatty acid oxidation in the liver and subsequent ketogenesis.

In contrast, diets rich in fatty acid and low in carbohydrate content, often referred to as ketogenic diets, promote ketogenesis as opposed to attenuating it, since the low carbohydrate content limits the islet β‐cell insulin response and subsequent anti‐lipolytic actions. Although diets rich in protein can contribute to ketogenesis via increasing supply of ketogenic amino acids such as leucine and phenylalanine, excessive protein consumption may also divert amino acids toward gluconeogenesis, thereby reducing ketogenesis (Veldhorst et al., 2009). Besides immediate effects of meal ingestion on ketone metabolism, it has been demonstrated that adaptations can develop with sustained adherence to ketogenic dietary patterns. More specifically, a study conducted in mice maintained on a ketogenic diet for 5 weeks showed that long‐term adaptation to such a diet may result in a reduced capacity for ketone utilization in cardiac tissue. This effect was attributed to a reduction in *Oxct1* (the gene encoding succinyl‐CoA:3‐ketoacid‐CoA transferase; SCOT) expression, though whether this adaptation occurs in other organs remains to be determined.

## 4 KETONE OXIDATION IN BRAIN AND OTHER ORGANS

Circulating βOHB is transported into cells of its target organs (e.g., neurons of the brain, cardiomyocytes of the myocardium) via either passive diffusion or uptake through monocarboxylate transporters (MCTs), the latter being upregulated at the blood–brain barrier as the brain's demand for ketones increases (Courchesne‐Loyer et al., 2017; Lengacher et al., 2013). In neurons and other cell types with the capacity to oxidize ketones, βOHB is first converted back to acetoacetate by BDH1, which also produces the reducing equivalent NADH (Figure 1). Subsequently, the rate‐limiting enzyme of ketone oxidation in normal physiology, SCOT, transfers the CoA moiety from succinyl‐CoA to acetoacetate, producing acetoacetyl‐CoA, which is then converted to two molecules of acetyl‐CoA by ACAT (Figure 1). Similar to oxidative metabolism of fatty acids and carbohydrates, this acetyl‐CoA shuttles toward the Krebs cycle, leading to the generation of reducing equivalents (e.g., NADH) that donate their electrons to the complexes of the electron transport chain to support oxidative phosphorylation and subsequent ATP production. Ketone uptake in the brain is closely correlated with the circulating concentrations; thus, as serum ketone levels rise, such as is observed during prolonged fasting/starvation, cerebral ketone uptake and subsequent oxidation increase in a linear fashion (Bennett et al., 2022; Courchesne‐Loyer et al., 2017).

As previously stated, many other organs, such as the heart and skeletal muscle, are also avid consumers of ketones (Puchalska & Crawford, 2017; Robinson & Williamson, 1980; Soni et al., 2024). In particular, due to comprising ∼40% of total body mass, skeletal muscle is an important consumption site for ketones, such that changes in muscle ketone metabolism/oxidation can significantly impact circulating ketone levels (Cotter et al., 2013). Furthermore, increases in circulating ketone levels and subsequent ketone oxidation have been shown to decrease muscle glycolysis rates while also preserving muscle glycogen content (Cox et al., 2016). In contrast, changes in cardiac ketone metabolism/oxidation are unlikely to robustly influence circulating ketone levels, but on a per gram basis, the heart is the largest consumer of ketones as an oxidative fuel source (Lopaschuk & Dyck, 2023). In addition, an infusion of βOHB leads to a decrease in fatty acid oxidation rates in the pig heart as assessed using [^3^H]palmitate (Stanley et al., 2003), whereas cardiac‐specific knockout (KO) of either SCOT or BDH1 in mice results in a corresponding elevation in fatty acid oxidation as assessed with nuclear magnetic resonance spectroscopy using [U‐^13^C]palmitate (Horton et al., 2019; Schugar et al., 2014). In contrast, studies in isolated working mouse hearts using [3‐^14^C]βOHB observed that increases in perfusate βOHB levels and subsequent ketone oxidation rates have no impact on cardiac fatty acid or glucose oxidation rates (Ho et al., 2021).

The kidney has also been demonstrated to be an avid consumer of ketones, whereby βOHB derived ATP production has been shown to enhance renal oxygenation (Mulder et al., 2019). Furthermore, the adaptive response of renal metabolism toward ketone utilization may contribute to maintaining glomerular filtration rate and supporting tubular reabsorption processes (Trevisan et al., 1987). Lastly, islets of the pancreas can also oxidize ketones, whereby it has been observed that increased βOHB oxidation mildly stimulates insulin secretion, likely via increasing β‐cell ATP levels and subsequent closure of ATP‐sensitive potassium channels, resulting in membrane depolarization (Albers et al., 2010).

## 5 KETONE METABOLISM IN RESPONSE TO EXERCISE

Prolonged sessions of endurance exercise are characterized by post‐exercise ketosis, wherein circulating ketone levels remain elevated for several hours following exercise (Johnson et al., 1969). Hepatic glycogen is depleted with prolonged exercise as the liver mobilizes glycogen to release glucose into the circulation for oxidation in the brain and in the muscle (Coker et al., 1997). Moreover, the rise in catecholamines during exercise increases adipose tissue lipolysis and subsequent circulating fatty acid levels (Galbo et al., 1975). As such, elevated fatty acid delivery, uptake and β‐oxidation in the liver significantly increases production of acetyl‐CoA, which is shuttled toward ketogenesis due to limited oxaloacetate availability needed for citrate formation through citrate synthase (Puchalska & Crawford, 2017; Robinson & Williamson, 1980). In rats, however, the extent of post‐exercise ketosis is reduced with endurance exercise training, as 6 weeks of treadmill training for 1 h twice a day reduced the extent of glycogen depletion following exercise (Beattie & Winder, 1984). In addition, 7 weeks of endurance exercise training (40 min/day; 6 days/week) also reduced the spike in circulating noradrenaline and adrenaline levels, thereby limiting the surge in hepatic fatty acid delivery to support β‐oxidation derived acetyl‐CoA production (Fery & Balasse, 1986; Winder et al., 1978).

The rise in circulating ketones during prolonged exercise or post‐exercise ketosis may lead to an increase in ketone oxidation in the brain, as ketone utilization rates are largely dictated by their circulating concentrations (Owen et al., 1967). Moreover, 12 weeks of endurance exercise training in rats (2 h/day; 5 days/week) increases the expression of BDH1 in the brain (Winder et al., 1974), though no studies to date have directly measured ketone oxidation in the brain during exercise. Again, due to the overall contribution to total body mass of skeletal muscle, increases in skeletal muscle ketone oxidation account for the majority of the exercise‐mediated increases in whole‐body ketone utilization (Balasse et al., 1978; Fery & Balasse, 1986). 14 weeks of treadmill training (90 min/day; 3 days/week) increases ketone uptake in rat hindlimb muscle (Ohmori et al., 1990). Furthermore, 12 weeks of treadmill training (5 days/week) also increases ketone utilization in rats, which is associated with increased expression of BDH1, SCOT and ACAT1 in skeletal muscle (Winder et al., 1974, 1975). This upregulation may be mediated by peroxisome proliferator‐activated receptor γ coactivator 1‐α (PGC‐1α), as skeletal muscle‐specific KO of PGC‐1α in mice blunts the increased expression of these enzymes in response to 8 weeks of exercise training when the mice are housed in cages with free access to voluntary run wheels (Svensson et al., 2016). Conversely, transgenic overexpression of PGC‐1α exacerbates the increased expression of ketone oxidation enzymes in the skeletal muscles of mice housed in cages with free access to voluntary run wheels for 8 weeks (Svensson et al., 2016). It should also be noted that expression levels of the enzymes of ketone oxidation in sedentary and exercised rats are dependent on muscle fibre type, with their expression being greatest in highly oxidative type I muscle fibres, followed by type IIa glycolytic fibres, with their lowest expression in type IIx glycolytic fibres (Winder et al., 1974). Of interest, this study observed that the greatest increase in ketone oxidation enzyme expression in response to exercise training occurred in type IIa glycolytic fibres. The general increase in skeletal muscle ketone oxidation enzyme expression is likely an adaptive response to exercise, thereby allowing the muscle to utilize the increased ketone supply it is being exposed to (Cox & Clarke, 2014).

In a study of 9 elite athletes, exogenous supplementation with the ketone ester (*R*)‐3‐hydroxybutyl (*R*)‐3‐hydroxybutyrate enhanced performance in a 30‐min cycling time trial versus placebo (Cox et al., 2016). Intramuscular triacylglycerol content from muscle biopsies of the individuals who received the ketone ester was significantly depleted following the time trial, whereas intramuscular glycogen content was only slightly depleted. This suggests that increased utilization of ketones during exercise also leads to a shift in substrate preference.

Ketones also play a pivotal role in recovery from exercise due to their glucose‐ and muscle protein‐sparing effects. In humans, exogenous supplementation with the ketone ester (*R*)‐3‐hydroxybutyryl (*R*)‐3‐hydroxybutyrate in combination with continuous glucose infusion, enhances glycogen replenishment in skeletal muscle following a glycogen‐depleting exercise protocol entailing a 12 h fast and an interval session to exhaustion on a stationary bike (Holdsworth et al., 2017). Furthermore, infusion of βOHB (∼1.2 mM) reduces circulating alanine and leucine levels (Nair et al., 1988; Sherwin et al., 1975), suggesting that ketones facilitate recovery in skeletal muscle by favouring protein synthesis over degradation.

## 6 KETONES AS REGULATORS OF CELL SIGNALLING

**FIGURE 2:**
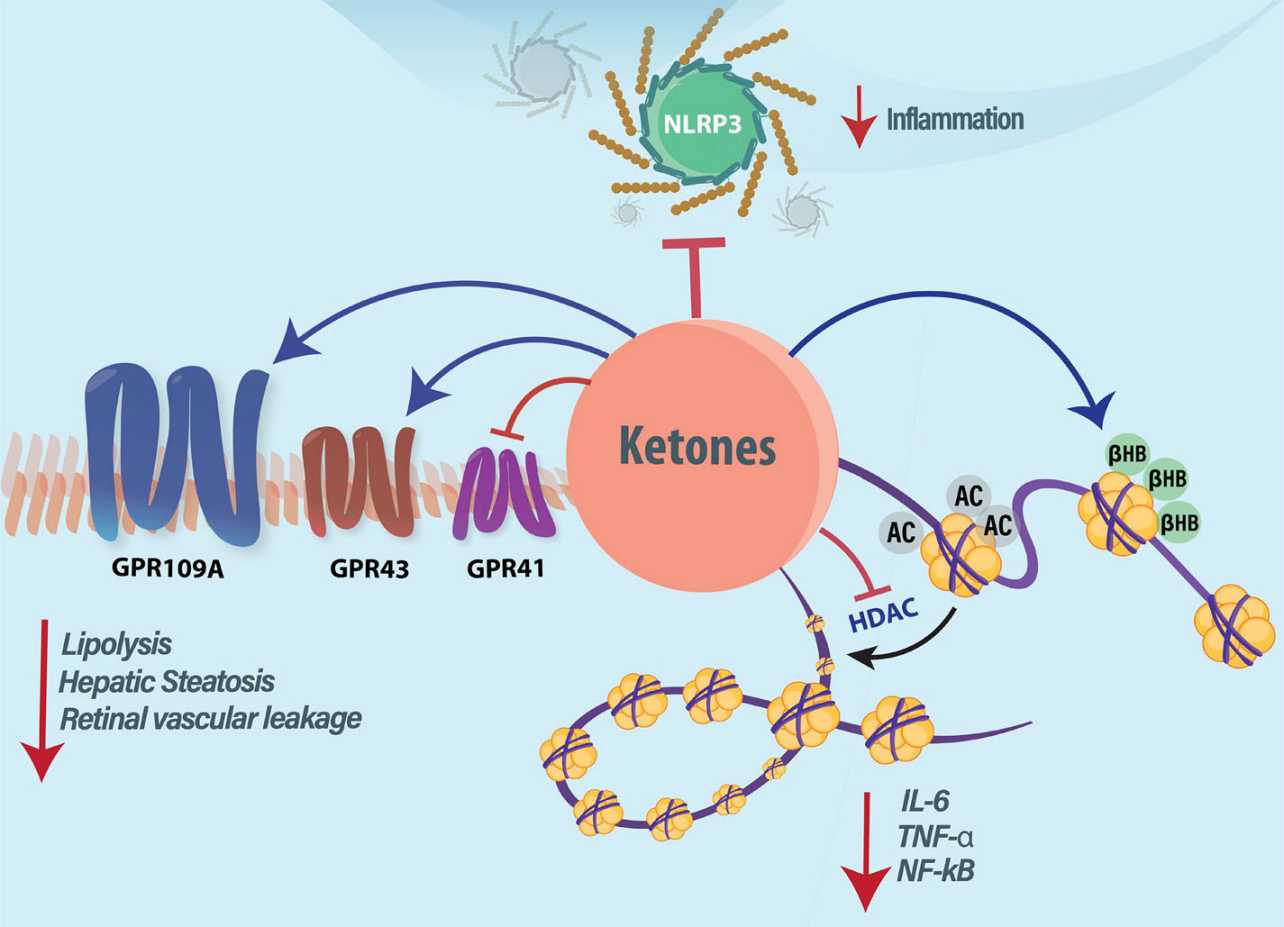
Ketone‐regulated cell signalling. Ketones modulate multiple cellular signalling pathways, exerting diverse physiological effects. By activating GPCRs, specifically GPR109A, GPR43 and GPR41, ketones suppress lipolysis and hepatic steatosis. Additionally, they promote gene expression either by inhibiting HDAC or through β‐hydroxybutyrylation. Ketones also mitigate inflammation by inhibiting the NLRP3 inflammasome. AC, acetylation; βHB, β‐hydroxybutyrylation; GPCRs, G protein‐coupled receptors; HDAC, histone deacetylases; NLRP3, NLR family pyrin domain containing 3.

As previously stated, ketones are more than just substrates supporting ATP production, as ketones (primarily βOHB) are also important signalling molecules. They are now recognized as ligands for several GPCRs, capable of modulating gene expression through direct and indirect mechanisms, while dampening inflammatory responses (Figure 2). The signalling actions ascribed to ketones discussed herein have been shown to influence various physiological processes, including metabolism, inflammation, and cardiac function, which have been extensively reviewed by others (Lopaschuk & Dyck, 2023; Puchalska & Crawford, 2017; Soni et al., 2024).

### 6.1 Ketones and GPCRs

One of the key GPCRs associated with ketone signalling is GPR109A, also known as hydroxy‐carboxylic acid receptor 2. A study utilizing isolated adipocytes from C57BL/6J mice demonstrated that activation of GPR109A using βOHB at very high doses (20 mM) leads to an inhibition of lipolysis (Taggart et al., 2005). As GPR109A is predominantly expressed in adipocytes and immune cells, this suggests a key link for ketone signalling in regulating immunometabolism. Furthermore, activation of GPR109A has been also shown to influence other aspects of immune cell function, particularly in macrophages, whereby it promotes a neuroprotective phenotype and reduced inflammation. Evidence of this was observed with βOHB infusion via subcutaneous mini pumps to maintain circulating βOHB levels at ∼1 mM, which reduced infarct size in wild‐type mice but not in mice with a macrophage‐specific GPR109A KO following middle cerebral artery occlusion (Rahman et al., 2014). In mice with streptozotocin‐induced type 1 diabetes, intraperitoneal administration of βOHB (700 mg/kg once daily for 8 weeks) reduced retinal vascular leakage in a GPR109A‐dependent manner (Abdelrahman et al., 2022). In addition to preserving endothelial function, treatment with βOHB (100 mg/kg for 30 days) in aged rats enhanced 5′AMP‐activated protein kinase activity, reduced protein expression of endoplasmic reticulum stress markers, circulating triacylglycerol levels and hepatic lipid content, which was associated with reduced mRNA expression of genes regulating lipogenesis (Lee et al., 2020). Illustrating that these actions may relate to GPR109A, treatment of HepG2 cells with βOHB (200–400 µM) for up to 6 h reduced endoplasmic reticulum stress and lipid accumulation, observations that were abolished via siRNA‐mediated knockdown of GPR109A.

Other GPCRs involved in ketone signalling include GPR41 and GPR43, which have been shown to interact not only with short‐chain fatty acids but also with ketones (Kimura et al., 2011). βOHB (but not acetoacetate) has been identified as an antagonist of GPR41 in HEK293 cells expressing mouse GPR41 (1 mM) and in cultured sympathetic neurons (10 mM) (Kimura et al., 2011). Treatment with βOHB (10 mM) has also been observed to modulate N‐Type calcium channels in sympathetic neurons isolated from adult male Wistar rats through activation of GPR41, though how this may impact neuronal function in vivo remains unknown (Won et al., 2013). Lastly, acetoacetate has been reported to act as an endogenous ligand for GPR43, decreasing intracellular cAMP levels in both GPR43 overexpressing HEK293 cells (at 0.3 mM) and in mouse embryonic fibroblast‐derived adipocytes (0.5–1.0 mM). (Miyamoto et al., 2019).

### 6.2 Ketones and gene expression

Several studies also support that ketones can influence gene expression, which may be mediated via epigenetic regulation secondary to modulating histone deacetylases (HDACs), essential enzymes controlling the acetylation status of histones (Falkenberg & Johnstone, 2014; Milazzo et al., 2020). The dynamic regulation of histone acetylation via HDAC plays an integral role in cell differentiation, proliferation, apoptosis and adaptive responses to environmental stimuli. Treatment with βOHB (1–32 mM) for 8 h increased histone acetylation levels in a dose‐dependent manner, as shown by elevated protein expression of acetylated histone H3 lysine residues 9 and 14 (Reichert et al., 2012). It should also be noted that βOHB appears to selectively inhibit class I HDACs without affecting HDAC6‐mediated tubulin deacetylation (Newman & Verdin, 2014; Shimazu et al., 2013).

Among short‐chain fatty acids, butyrate, which is structurally related to βOHB with the absence of a hydroxyl group, demonstrated greater potency as a HDAC inhibitor at equimolar dosing (4 mM) when compared to other short‐chain fatty acids (Newman & Verdin, 2014; Shimazu et al., 2013). Of interest, studies in HEK293 cells (10–40 mM βOHB for 18 h), HMEC‐1 cells (up to 20 mM ‐βOHB for 8 or 18 h) and rat L6 myotubes (40 mM βOHB for 1–24 h) did not observe inhibition of HDAC and subsequent histone acetylation status, even at physiologically implausible concentrations, whereas butyrate at 5 mM induced significant histone acetylation (Chriett et al., 2019). Hence, there appears to be a discrepancy regarding the ability of βOHB to inhibit HDACs, which may be dependent on cell type and supra‐pharmacological concentrations that can only be achieved in experimental settings.

Although the exact molecular mechanisms by which βOHB and short‐chain fatty acids bind/inhibit HDAC remain to be fully elucidated, recent investigations suggest that βOHB may also regulate gene expression via promoting an alternative post‐translational modification, β‐hydroxybutyrylation. β‐Hydroxybutyrylation alters the interaction between histones and DNA, leading to changes in chromatin architecture and influencing gene expression profiles more broadly than what is achieved through histone acetylation alone. Evidence of this was first demonstrated by detection of isotopically labelled β‐hydroxybutyryl‐CoA in a dose‐dependent manner, using high‐performance liquid chromatography–mass spectrometry, following treatment of HEK293 cells with isotopically labelled [2,4‐^13^C₂]βOHB (1–10 mM) (Xie et al., 2016; Zhou et al., 2022). Histone β‐hydroxybutyrylation specifically on histone 3, lysine 9 (H3K9) has been observed to modulate the expression of many genes, including *BDNF*, *VEGF*, *FOXO1*, *PPARGC1A* and *ADIPOQ*, promoting resilience to cellular stress (Chen et al., 2017; Zhang et al., 2020). It may be surmised that βOHB‐mediated β‐hydroxybutyrylation is the more relevant post‐translational modification by which ketones impact gene expression, since it occurs at βOHB levels typically reached during fasting or with adherence to ketogenic diets, unlike the higher concentrations required for HDAC inhibition.

### 6.3 Ketones and inflammation

Ketones have also been demonstrated to interact with the NLR family pyrin domain containing 3 (NLRP3) inflammasome, thereby mediating anti‐inflammatory actions. The NLRP3 is a multi‐protein complex, located in the cytosol of immune cells (primarily macrophages), serving as a guardian of the innate immune response. Treatment with βOHB (0.1–10 mM) decreased interleukin (IL)‐1β and IL‐18 levels in a dose‐dependent manner in isolated CD14^+^ monocytes from 6 healthy subjects stimulated with lipopolysaccharide (LPS; 1 µg/mL for 4 h) (Youm et al., 2015). Furthermore, in mouse bone marrow‐derived macrophages, treatment with 10 mM βOHB, but not butyrate or acetoacetate, inhibited NLRP3 activation as determined by reduced ATP‐induced cleavage of caspase‐1 and processing of IL‐1β. It has also been reported that βOHB prevents NLRP3 activation by inhibiting potassium efflux and reducing apoptosis‐associated speck‐like protein containing a caspase recruitment domain oligomerization and speck formation independent of GPR109A. Adherence to a ketogenic dietary pattern also alleviates NLRP3 activity, whereby in 15 healthy humans fed an isocaloric ketogenic diet for 3 days, decreased IL‐1β secretion was observed in ATP‐ or palmitate‐stimulated isolated human macrophages (Kim et al., 2022). However, whether this is due to the elevation of circulating ketones or some other aspect of the ketogenic diet remains unknown.

Contrasting studies in healthy individuals (10 males and 10 females) consuming ketone supplements have reported opposing findings (Neudorf et al., 2019). Acute ketone monoester ((*R*)‐3‐hydroxybutyl (*R*)‐3‐hydroxybutyrate) ingestion increased IL‐1β and IL‐6 levels following stimulation with LPS in whole blood supernatants, but did not result in changes in tumour necrosis factor‐α (TNF‐α) or IL‐8 levels. It has also been demonstrated that acute ingestion of (*R*)‐3‐hydroxybutyl (*R*)‐3‐hydroxybutyrate notably increases caspase‐1 activation as determined by flow cytometry, without affecting TNF‐α or IL‐8 levels. Despite these interesting findings, further investigation in both animals and humans is needed to determine whether the effects of acute versus chronic elevations in circulating βOHB levels extend beyond LPS‐induced inflammation, and to clarify their role in regulating chronic inflammation associated with several cardiometabolic diseases (e.g., T2D, heart failure).

## 7 TARGETING KETONE METABOLISM TO TREAT CARDIOMETABOLIC DISEASE

The field's appreciation of metabolism of ketones now extends beyond their serving as an alternative fuel source for the brain during prolonged/fasting or starvation. Of clinical interest, it is now becoming clear that perturbations in ketone metabolism contribute to the pathologies of several cardiometabolic diseases. The ensuing sections will describe whether targeting ketone metabolism may have clinical utility in the management of both T2D and heart failure (Table 1).

### 7.1 Ketone metabolism and T2D

Several studies support that perturbations in skeletal muscle ketone oxidation may contribute to insulin resistance and/or T2D. In a study involving 47 lean and 47 age‐matched obese women, a decrease in βOHB oxidation rates was observed in rectus abdominus muscle biopsy homogenates from the obese participants (Vice et al., 2005). While this may suggest on the surface that obesity impairs skeletal muscle ketone oxidation, this decrease was likely related to a marked reduction in mitochondrial content as reflected by a 50% decline in citrate synthase activity. Conversely, it has been demonstrated that obesity can also lead to potential increases in skeletal muscle ketone oxidation, as mRNA/protein expression and enzyme activity of SCOT are elevated in gastrocnemius muscles from obese male C57BL/6J mice (Al Batran et al., 2020). This potential elevation in skeletal muscle ketone oxidation is likely a maladaptive response during the progression of obesity‐related insulin resistance/T2D, as skeletal muscle‐specific KO of SCOT and subsequent ketone oxidation in mice led to robust improvements in glycaemia and glucose tolerance in response to experimental obesity/T2D (Al Batran et al., 2020). Intriguingly, these observations could be replicated via pharmacological means, as in silico modelling studies identified that the diphenylbutylpiperidine drug class of antipsychotics (e.g., pimozide, penfluridol) are capable of inhibiting SCOT activity, which also led to improved glycaemia in obese male mice (Tabatabaei Dakhili, Greenwell et al., 2023). Conversely, insulin‐stimulated Akt phosphorylation and 2‐deoxyglucose uptake were decreased in isolated mouse soleus muscles incubated with 5 mM βOHB, once again supporting that increases in skeletal muscle ketone metabolism may have harmful actions on glycaemia (Ivarsson et al., 2011).

Although it remains unknown why decreasing skeletal muscle ketone oxidation would protect against insulin resistance/T2D, the observed metabolic benefit may simply relate to substrate competition, whereby limiting ketone utilization leads to a corresponding increase in carbohydrate utilization. As evidence of this, pimozide‐mediated SCOT inhibition failed to alleviate obesity‐induced hyperglycaemia in mice with an inability to metabolize carbohydrates in their muscle secondary to a skeletal muscle‐specific KO of pyruvate dehydrogenase (Al Batran et al., 2020). While this may contribute to how restricting skeletal muscle ketone oxidation can improve glycaemia during insulin resistance/T2D, it is likely that other mechanisms are also involved. In particular, increases in circulating ketone levels secondary to treatment with ketone esters, which would increase skeletal muscle ketone delivery and subsequent oxidation, also improve glycaemia in obese mice (Tabatabaei Dakhili, Yang et al., 2023). These observations have been replicated in several studies in mice and even humans with obesity/T2D (Falkenhain et al., 2022; Soto‐Mota et al., 2021; Thai et al., 2021; Walsh et al., 2021). However, these salutary actions were still observed in skeletal muscle‐specific SCOT KO mice, demonstrating that the mechanism by which increasing circulating ketone levels improves glycaemia in obesity is independent from their metabolism/oxidation in skeletal muscle (Tabatabaei Dakhili, Yang et al., 2023). This suggests that the signalling responses impacted by ketones also contribute to their glucose‐lowering actions, though it remains to be determined whether this directly relates to the signalling responses ascribed to ketones in the previous section (Section 6). Taken together, both decreasing skeletal muscle ketone oxidation and increasing ketone delivery to the skeletal muscle (and subsequent oxidation) impart benefit on glycaemia. Despite studies suggesting that the former improves glycaemia via influencing substrate competition (increased glucose metabolism), both approaches would lead to the accumulation of βOHB within muscle and the surrounding microvasculature, suggesting that ketone‐mediated signalling is the predominant mechanism at play.

### 7.2 Ketone metabolism and heart failure

In 2016, concurrent studies in both animals and humans reported that heart failure was associated with increased myocardial ketone utilization rates (Aubert et al., 2016; Bedi et al., 2016), leading many in the field to posit that this observed increase was an adaptive response to account for heart failure‐mediated declines in fatty acid utilization. Supporting this conjecture, decreased cardiac ketone oxidation rates in cardiac‐specific SCOT KO mice are associated with worsened cardiovascular outcomes (e.g., decreased left ventricular ejection fraction (LVEF), ventricular wall thinning) in response to transverse aortic constriction (TAC)‐induced heart failure (Schugar et al., 2014). Mice with a cardiac‐specific BDH1 KO also demonstrated worsened cardiovascular outcomes (e.g., decreased LVEF) following TAC‐induced heart failure, whereas a 2‐week continuous infusion of βOHB into the right ventricle prevented the decline of LVEF and cardiac output in a tachypacing model of heart failure in canines (Horton et al., 2019). Likewise, increased cardiac ketone oxidation rates in mice with a cardiac‐specific overexpression of BDH1 led to improved cardiovascular outcomes (e.g., increased LVEF, decreased cardiac hypertrophy) in response to TAC‐induced heart failure (Uchihashi et al., 2017). In rat studies, increasing circulating ketones via incorporating either a hexanoyl‐hexyl‐βOHB ketone ester into the diet (10 or 15% w/w) or a βOHB‐butanediol monoester into standard chow improved LVEF and decreased cardiac hypertrophy following permanent left anterior descending coronary artery ligation‐induced heart failure (Yurista et al., 2021). Using a genetic approach, increases in circulating ketones secondary to skeletal muscle‐specific KO of SCOT in mice also resulted in improved cardiovascular outcomes (e.g., increased LVEF, decreased cardiac hypertrophy, decreased cardiac brain natriuretic peptide mRNA expression) following TAC‐induced heart failure (Byrne et al., 2020).

In contrast, increasing circulating ketone levels and subsequent cardiac ketone oxidation rates with ketogenic diets did not improve cardiac function and decreased cardiac efficiency in mice subjected to ischaemic heart failure via permanent occlusion of the left anterior descending coronary artery (Ho et al., 2024). While such observations may suggest that increasing cardiac ketone oxidation rates do not benefit cardiac function in heart failure, it is important to note that the high‐fat composition of ketogenic diets may explain these specific outcomes, considering that fatty acids have negative actions on cardiac efficiency (Lopaschuk et al., 2010). Nonetheless, other studies have suggested that decreasing cardiac ketone oxidation can be beneficial in the setting of diabetic cardiomyopathy, which is a condition characterized by diastolic dysfunction that can increase risk for heart failure in diabetes (Heather et al., 2024; Ritchie & Abel, 2020). More specifically, isolated working heart studies demonstrated that cardiac ketone oxidation rates are decreased in mice with diabetic cardiomyopathy (Greenwell et al., 2024). Intriguingly, pharmacological inhibition of SCOT activity and subsequent cardiac ketone oxidation rates via treatment with pimozide in mice with diabetic cardiomyopathy led to an improvement in diastolic function, while decreasing left atrial enlargement and cardiomyocyte hypertrophy.

Several studies have now reported the actions of increasing circulating ketones in humans with heart failure. In 12 individuals with heart failure with reduced ejection fraction (LVEF < 40%) participating in a crossover study, an oral dose of (*R*)‐1,3‐butanediol increased circulating ketones over 6 h, which was associated with increased stroke volume and LVEF versus when they ingested placebo (Guldbrandsen et al., 2025). Moreover, in 36 individuals with heart failure with reduced ejection fraction (LVEF < 50%) and T2D, it was observed that infusion with βOHB for 6 h aimed at maintaining βOHB levels at 0.7, 1.6 or 3.2 mM led to significant increases in stroke volume and LVEF in the latter (Solis‐Herrera et al., 2025). In a crossover study completed by 24 individuals with heart failure with preserved ejection fraction and T2D, 2 weeks of treatment with d‐βOHB‐l‐1,3‐butanediol (25 g, 4 times a day) produced salutary actions on haemodynamic parameters (increased cardiac output, decreased pulmonary capillary wedge pressure) at rest and during exercise (Gopalasingam et al., 2024). Intriguingly, the sodium–glucose co‐transporter 2 (SGLT2) inhibitors, which are an antidiabetic drug class that have been demonstrated to improve cardiovascular outcomes in people with T2D, often lead to increases in circulating ketone levels (Lopaschuk & Dyck, 2023; Puchalska & Crawford, 2017). It has been posited that the beneficial actions of SGLT2 inhibitors on cardiovascular outcomes in T2D are dependent on increases in cardiac ketone oxidation, though this remains an ongoing topic of debate (Lopaschuk & Verma, 2020; Packer, 2023). More recent studies suggest that an SGLT2 inhibitor‐mediated increase in cardiac ketone oxidation is unlikely to be the mechanism at play, as cardiac energetics were unchanged following empagliflozin treatment in the ‘Assessment of Cardiac Energy Metabolism, Function and Physiology in Patients with Heart Failure Taking Empagliflozin’ study (Hundertmark et al., 2023). Nonetheless, this does not rule out that SGLT2 inhibitors improve cardiovascular outcomes secondary to impacting ketone‐mediated signalling.

In general, the vast majority of experimental studies in animals support that increasing circulating ketones does appear to have beneficial actions for the failing heart. Likewise, this has been, for the most part, recapitulated in humans with heart failure, though it remains unknown whether this relates to increases in cardiac ketone oxidation per se, or to the several signalling responses directly impacted by ketones. As most of the beneficial studies have employed the various ketone esters or direct administration of βOHB as their approach to increase circulating ketones, further research is needed to determine whether adherence to ketogenic diets will yield similar actions in heart failure.

## 8 SUMMARY, CONCLUSIONS AND FUTURE CONSIDERATIONS

From the original misconception of being wasteful products of incomplete fatty acid oxidation, to recognition of being a major fuel source for the brain during prolonged fasting/starvation, the field's appreciation of ketones and their essential roles in whole‐body metabolic homeostasis continues to grow. This includes appreciation of the key role for ketones in supporting whole‐body metabolism during exercise, and the recognition that ketones serve as ligands for GPCRs to impact cell signalling. Because ketones have widespread importance in whole‐body metabolic homeostasis, it comes as no surprise that perturbations in ketone metabolism can contribute to the pathologies of several cardiometabolic diseases, including T2D and heart failure. While a role for targeting ketone metabolism in both T2D and heart failure remains inconclusive, an area of particular importance surrounds whether any potential benefit to manipulating ketone metabolism involves its role in supporting ATP production, or rather, to serve as a regulator of cell signalling. With how rapidly this field has been evolving, the answers to these questions should become clearer sooner rather than later, thus guiding whether pharmacological or nutritional strategies to manipulate ketone metabolism have a future role in the management of T2D, heart failure or other cardiometabolic diseases.

## AUTHOR CONTRIBUTIONS

All authors contributed to writing the original draft of this manuscript, as well as editing its final version. All authors approved the final version of the manuscript, while agreeing to be accountable for all aspects of the work relating to its accuracy and/or integrity. Lastly, all individuals designated as authors qualify for authorship, and all those who qualify for authorship are listed.

## CONFLICT OF INTEREST

None declared.
